# The frequency of atrial fibrillatory waves is modulated by the spatiotemporal pattern of acetylcholine release: a 3D computational study

**DOI:** 10.3389/fphys.2023.1189464

**Published:** 2024-01-03

**Authors:** Chiara Celotto, Carlos Sánchez, Mostafa Abdollahpur, Frida Sandberg, Jose F. Rodriguez Mstas, Pablo Laguna, Esther Pueyo

**Affiliations:** ^1^ BSICoS Group, I3A and IIS-Aragón, University of Zaragoza, Zaragoza, Spain; ^2^ CIBER - Bioingeniería, Biomateriales, y Nanomedicina (CIBER-BBN), Zaragoza, Spain; ^3^ Department of Biomedical Engineering, Lund University, Lund, Sweden; ^4^ Department of Chemical and Material Engineering, Politecnico Di Milano, Milan, Italy

**Keywords:** atrial fibrillation, f-waves, acetylcholine, computational simulation, cardiorespiratory modulation, autonomic nervous system

## Abstract

In atrial fibrillation (AF), the ECG P-wave, which represents atrial depolarization, is replaced with chaotic and irregular fibrillation waves (f waves). The f-wave frequency, *F*_f_, shows significant variations over time. Cardiorespiratory interactions regulated by the autonomic nervous system have been suggested to play a role in such variations. We conducted a simulation study to test whether the spatiotemporal release pattern of the parasympathetic neurotransmitter acetylcholine (ACh) modulates the frequency of atrial reentrant circuits. Understanding parasympathetic involvement in AF may guide more effective treatment approaches and could help to design autonomic markers alternative to heart rate variability (HRV), which is not available in AF patients. 2D tissue and 3D whole-atria models of human atrial electrophysiology in persistent AF were built. Different ACh release percentages (8% and 30%) and spatial ACh release patterns, including spatially random release and release from ganglionated plexi (GPs) and associated nerves, were considered. The temporal pattern of ACh release, ACh(*t*), was simulated following a sinusoidal waveform of frequency 0.125 Hz to represent the respiratory frequency. Different mean concentrations $\left(\bar{ACh}\right)$ and peak-to-peak ranges of ACh (ΔACh) were tested. We found that temporal variations in *F*_f_, *F*_f_(*t*), followed the simulated temporal ACh(*t*) pattern in all cases. The temporal mean of *F*_f_(*t*), ${\bar{F}}_{\text{f}}$ , depended on the fibrillatory pattern (number and location of rotors), the percentage of ACh release nodes and $\bar{ACh}$ . The magnitude of *F*_f_(*t*) modulation, Δ*F*_f_, depended on the percentage of ACh release nodes and ΔACh. The spatial pattern of ACh release did not have an impact on ${\bar{F}}_{\text{f}}$ and only a mild impact on Δ*F*_f_. The f-wave frequency, being indicative of vagal activity, has the potential to drive autonomic-based therapeutic actions and could replace HRV markers not quantifiable from AF patients.

## 1 Introduction

 The heart rhythm is controlled by a multilevel neural network with involvement of the central nervous system and peripheral autonomic nervous system (ANS), the latter exerting its activity through the sympathetic and parasympathetic branches (Shivkumar et al., 2016). The intrinsic cardiac nervous system is organized in clusters of autonomic ganglia called ganglionated plexi (GPs) and in an epicardial neural network of local circuit neurons that work as inter- and intra-ganglionic connections. The role of the GPs is to modulate cardiac electrophysiology, acting as a local hub to integrate the inputs from the epicardial neural network and from the extrinsic innervation. The activation of efferent neurons acts to modulate the heart rate, the atrioventricular node conduction and the inotropism of atria and ventricles (Fedele and Brand, 2020).

It has been shown that ANS activity is closely related to the genesis and maintenance of atrial arrhythmias, including atrial fibrillation (AF) (Chang et al., 2016). While sympathetic activation mainly acts as a trigger by facilitating the generation of ectopic beats, vagal hyperactivity facilitates the formation of reentries by shortening the wavelength of reentry (WL), defined as the distance traveled by the depolarization wave during the effective refractory period (ERP) (Rohr et al., 1998) and estimated as the product of the CV and ERP. In fact, acetylcholine (ACh), the neurotransmitter released by pre- and postganglionic parasympathetic nerve terminals, shortens action potential (AP) duration (APD) and hyperpolarizes the resting membrane potential (RMP) in a dose-dependent manner. Hyperpolarization of the RMP leads to a reduction in the maximum upstroke velocity of the AP (Rohr et al., 1998), which can also slow CV.

During AF, the P-waves of the electrocardiogram (ECG), representative of atrial activation, are replaced with a series of waves known as fibrillatory waves (f waves) (Sörnmo et al., 2018). Some f-wave features have been proposed to characterize atrial electrical activity during AF, including the amplitude, morphology, regularity, complexity and frequency (Petrutiu et al., 2006; Meo et al., 2013; Lankveld et al., 2014; Sörnmo et al., 2018. Among those features, the f-wave frequency (*F*_f_), often referred to as the atrial fibrillatory rate, has received considerable clinical attention (Lankveld et al., 2014; Platonov et al., 2014; Östenson et al., 2017). The mean f-wave frequency, also referred to as the atrial fibrillatory rate (AFR), has been associated with the complexity of arrhythmias. Lower AFR values have been linked to a higher likelihood of spontaneous cardioversion in patients with paroxysmal atrial fibrillation (pxAF) (Bollmann et al., 1999; Choudhary et al., 2013). Moreover, shorter AF episodes tend to exhibit lower AFR values compared to longer-lasting episodes, with AFR decreasing before termination (Bollmann et al., 1999). We believe that monitoring changes in the parameter *F*_f_ over time can provide valuable insights. Upon confirmation that the temporal variations in *F*_f_ offer indications of parasympathetic activity, they could potentially guide therapies aimed at modulating autonomic nervous system activity, which has long been recognized as an important contributing factor to proarrhythmia, and possibly replace HRV markers not quantifiable in AF. Different methods have been employed to compute *F*_f_. In some studies, *F*_f_ has been derived through spectral (frequency domain) analysis by identifying the frequency presenting the highest peak in the power spectral density (Park et al., 2019). In other works, *F*_f_ has been extracted from the analysis of the ECG in the time domain using a model-based approach (Henriksson et al., 2018; Abdollahpur et al., 2021).

 Previous studies have shown that *F*_f_ can be influenced by changes in the autonomic tone. For instance, *F*_f_ has been shown to increase in response to head-up tilt (Ingemansson et al., 1998) and decrease in response to head-down tilt (Ingemansson et al., 1998; Östenson et al., 2017). These tilt maneuvers are known to modulate the activity of the parasympathetic and sympathetic nervous systems. In sinus rhythm, cardiorespiratory interactions through the ANS have been widely studied (Yasuma and Hayano, 2004), with respiratory sinus arrhythmia (RSA) defined as the autonomically-mediated modulation of the sinus node pacemaker frequency in synchrony with inspiration and expiration (Hirsch and Bishop, 1981; Eckberg, 1983; Piepoli et al., 1997; Eckberg, 2000; Eckberg, 2003; Yasuma and Hayano, 2004). The contribution of RSA to heart rate variability (HRV) can be measured from the high-frequency components of HRV and can be used for noninvasive assessment of parasympathetic activity (Kunze, 1972; Katona and Jih, 1975; Eckberg, 1983; Piepoli et al., 1997; Eckberg, 2003). Associated with enhanced vagal activity (Masé et al., 2008). There is currently a need for non-invasive methods to assess ANS activity during AF other than HRV, which cannot be used to measure ANS activity during AF since heartbeats do not originate in the sinoatrial node (SAN). It is worth exploring the impact of differences in ANS activity among AF patients on their responses to treatment. The hyperactivity of the parasympathetic nervous system has been established as a mechanism that promotes the initiation of AF. However, this observation may apply to some patients but not to others. Abdollahpur et al. found that the respiratory modulation of f-waves was reduced in some patients after full vagal blockade, while in others, it remained unchanged (Abdollahpur et al., 2021). Being able to non-invasively assess vagal activity could guide clinicians towards one treatment approach over another, whether it be pharmacological therapy, cardioneuroablation, or neural modulation treatments. However, there is limited research on how respiration affects atrial rate during atrial tachyarrhytmias, particularly AF, primarily due to technical difficulties (Holmqvist et al., 2005). These difficulties include distinguishing noise from f-waves, the presence of modulation that is unrelated to the respiratory signal, the very small magnitude of the respiratory-induced f-wave frequency modulation that may be concealed by other variations and the unknown and variable respiration rate over time. The study by Abdollahpur et al. (2022), Respiratory induced f-wave frequency variations were observed at baseline and during deep breathing. The study by Holmqvist et al. and the follow-up by Abdollahpur et al. (2021) were, however, able to show that low frequency-controlled respiration can induce cyclic fluctuations in *F*_f_, which may be linked to parasympathetic regulation of the atrial WL, given that the modulation was reduced in response to vagal blockade.

 We used computational modeling and simulation to evaluate the role of the spatiotemporal release pattern of ACh, considered to temporally vary in phase with inspiration and expiration, in the modulation of the f-wave frequency. Both two-dimensional (2D) tissues and three-dimensional (3D) whole-atria models representative of persistent AF (psAF) were built and distinct spatial distributions of ACh release sites were defined in the models. Also, different stimulation protocols were simulated to evaluate the role of the spatiotemporal ACh release pattern combined with the reentry characteristics on the fibrillatory rate. To provide a more extensive characterization, *F*_f_ was analyzed in terms of mean $\left({\bar{F}}_{f}\right)$ and range of variation (Δ*F*_f_), as studies on ECGs from patients have shown Δ*F*_f_ to provide complementary information to ${\bar{F}}_{f}$ (Abdollahpur et al., 2021). The computational results from the present work were compared with results obtained from the analysis of clinical data (Abdollahpur et al., 2021). The final objective was to assess the impact of vagal stimulation on the AF fibrillatory frequency to potentially assess differences in autonomic modulation of atrial activity in psAF patients.

## 2 Methods

### 2.1 Atrial models

 Human atrial electrical activity was simulated in 2D square sheets of tissue and in 3D biatrial anatomical models. The 2D models represented square pieces of tissue of 7 × 7 cm, discretized in square elements of 200 *μ*m side. A uniform bottom-to-top fiber direction was assigned to the tissues. For the 3D biatrial models, the anatomy was in all cases defined as in Ferrer et al. (2015). The 3D anatomical model was discretized using linear hexahedral elements with an average edge length of 300 *μ*m. The wall thickness varied between 600 and 900 *μ*m, resulting in 2 or 3 hexahedral elements arranged transversely. This resulted in a total of 754,893 nodes and 515,010 elements. The model included detailed regional descriptions of fiber direction and functional heterogeneity, considering eight regions with different electrophysiological properties.

 To simulate the electrophysiological activity of the cardiomyocytes, the Courtemanche human atrial AP model was used (Courtemanche et al., 1998). In the 2D models, all the myocardial nodes were assigned with the same electrophysiological characteristics representative of left atrial tissue. In the 3D models, the Courtemanche model was adapted to represent different atrial regions by varying the ionic current conductances as in Ferrer et al. (2015). These adjustments were made based on experimental observations regarding AP morphology and duration reported in several studies (Wang et al., 1990; Wang et al., 1993; Feng et al., 1998; Li et al., 2001; Cha et al., 2005; Seemann et al., 2006). The eight regions with different electrophysiological characteristics were: left atrium (LA), right atrium (RA), left atrial appendage (LAA), right atrial appendage (RAA), pulmonary veins (PV), tricuspid valve ring (TVR), mitral valve ring (MVR), crista terminalis and Bachmann bundle (CTBB). In Supplementary Figure S1, the APs corresponding to these regions are shown.

To model cholinergic stimulation, an ACh-activated potassium current (I_KACh_) was introduced in the AP model following the formulation developed by Kneller et al. (2002) and updated by Bayer et al. (2019), as in previous studies (Celotto et al., 2021; Celotto et al., 2023). The equation is reported below:

$$\begin{array}{ll} I_{\text{KACh}}\left(t\right) & =\left(\frac{10.0}{1+\frac{9.14}{{\left(ACh\left(t\right)10^{-1}\right)}^{0.478}}}\right)\left(0.05\right.+\left.\frac{5.0}{1+e^{\frac{V_{m}\left(t\right)+85.0}{5.0}}}\right)\\ & \;\left(V_{m}\left(t\right)-E_{k}\left(t\right)\right) \end{array}$$ (1)

where *ACh*(*t*) is the ACh concentration at time “t” expressed in *μ*M, *V*_*m*_(*t*) is the membrane potential and *E*_*k*_(*t*) is the Nernst potential for potassium.

Electrical remodeling associated with psAF was represented by altering the conductances of four ionic currents. The conductances of the transient outward potassium current (I_to_), the L-type calcium current (I_CaL_) and the ultrarapid delayed rectifier potassium current (I_Kur_) were reduced by 50%, 70% and 50%, respectively, as in Courtemanche (1999), and the conductance of the inward rectifier potassium current (I_K1_) was increased by 100% (Dobrev et al., 2001).

PsAF-induced structural remodeling was modeled by including 20% diffuse fibrosis on the basis of histological studies reporting diffuse fibrosis percentages up to 40%, with a mean of approximately 20%, in psAF patients (Platonov et al., 2011; Ma et al., 2021). Both in the 2D and 3D models, 20% of the nodes were uniformly randomly selected. These nodes were assigned with fibroblast properties and their electrical activity was defined by the MacCannell active fibroblast computational AP model (MacCannell et al., 2007). This model comprises a time- and voltage-dependent potassium current (I_Kv_), an inward rectifying potassium current (I_K1_), a sodium-potassium pump current (I_NaK_) and a background sodium current (I_bNa_) (MacCannell et al., 2007). The fibroblast-fibroblast gap-junctional conductance was reduced 4-fold with respect to the myocyte-myocyte conductance described in the following paragraph. Our approach aligns with previous computational studies, which simulated gap junction remodeling in AF-remodeled tissues by reducing the conductance to similar levels (Li et al., 1999; Burstein et al., 2009; McDowell et al., 2013; Krueger et al., 2014). When myocytes were coupled to fibroblasts, the junctional conductance was linearly adjusted depending on the number of fibroblasts coupled to a myocyte.

The myocyte-myocyte conductance was adapted to match experimental evidence in terms of CV and total activation time (TAT). In the 3D models, we used values of longitudinal conductivity (L_CV_) and transverse-to-longitudinal conductivity ratio (T/L_CR_) that were adapted from Ferrer et al. (2015) for different atrial regions. In their study, Ferrer et al. (2015) initially identified 21 anatomical atrial regions characterized by the same CV, on the base of anatomical and histological information. However, they found that only ten regions significantly influenced the body surface potential maps and the main features of the P-wave. Building upon their work, our study focused on these ten anatomical regions. Particularly, for definition of the conductivity values, we considered ten atrial regions: LA, RA, PV, SAN, coronary sinus (CS), isthmus (IST), the fossa ovalis (FO) and its limb (LFO), the crista terminalis (CT), the Bachmann bundle and the pectinate muscles (BBPM). The defined conductivity values, reported in Supplementary Table S1, led to a TAT of 180 ms, in line with data reported in the literature for psAF patients (Wesselink et al., 2022).

It is worth noting that the heterogeneities in electrophysiological characteristics and in conductivities were defined by using eight and ten atrial regions, respectively, so as to match experimental evidence. The characteristics defining tissue conductivity are in fact independent of the electrophysiological characteristics of the myocytes.

In the 2D models, we used the same values of longitudinal conductivity and transverse-to-longitudinal conductivity ratio as in the LA region of the 3D model, which rendered a longitudinal CV of 50 cm/s for a planar wave, in agreement with values reported for AF patients in previous studies (Bayer et al., 2019).

### 2.2 Simulated ACh release patterns

We defined four different 3D models corresponding to distinct spatial ACh release configurations throughout the atria. These models were based on different experimental and computational studies, resulting in two different volumetric percentages of ACh release sites (i.e., 8% and 30%). The ACh release was transmural, with ACh release nodes being found all throughout the transmural wall (except for the $O_{08}$ and $O_{30}$ models, in which ACh release nodes were restricted to the two most external layers).

**FIGURE 1:**
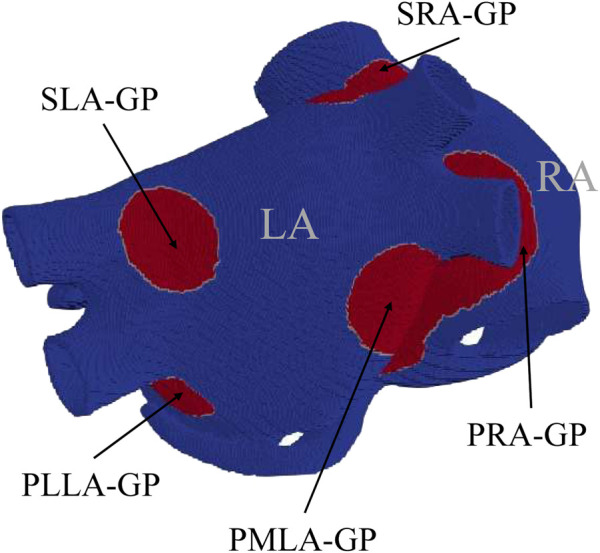
Computational model used in this study, with GPs represented in red, following the anatomical description by Armour et al. (1997).

**FIGURE 2:**
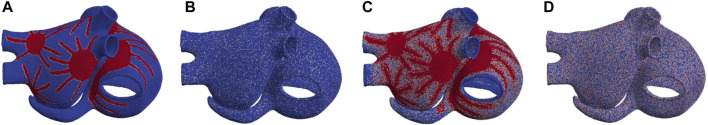
3D biatrial anatomies, with ACh release nodes depicted in red. Representation of **(A)** $O_{08}$ model, **(B)** $D_{08}$ model, **(C)** $O_{30}$ model and **(D)** $D_{30}$ models.

The base of our atrial models of ACh release is the experimental anatomical study by Armour et al. (1997), which mapped the major atrial and ventricular GPs. This study provided us with information on the location and size of GP cores, as represented in Figure 1. The following five major GPs were considered: the superior right atrial GP (SRA-GP), located on the posterior superior surface of the RA close to the junction of the SVC and RA; the superior left atrial GP (SLA-GP), located on the posterior surface of the LA between the PVs; the posterior right atrial GP (PRA-GP), located on the posterior surface of the RA adjacent to the inter-atrial groove; the posteromedial left atrial GP (PMLA-GP), located on the posterior medial surface of the LA; and the posterolateral left atrial GP (PLLA-GP), located on the posterior lateral surface of the LA base on the atrial side of the atrioventricular groove. To account for the communication of GPs with the atrial tissue, we integrated the octopus hypothesis based on prior experimental and computational studies (Zhou et al., 2007; Hwang et al., 2016). This hypothesis suggests that the GPs send out eight branches, each about 2 cm long (Hwang et al., 2016), representing the main nerves that branch off from them. Finally, we accounted for the transmural distribution of nerve plexuses. Considering that the neural cell distribution is mainly epicardial (Tan et al., 2006), we concentrated the ACh release mostly in the two more external layers, as represented in Supplementary Figure S2. The resulting model, denoted as $O_{08}$ , realistically represented the heterogeneous ACh release in the atria. Considering both the GP bodies and the nerves departing from them, 8% of all the mesh nodes resulted to be ACh release nodes. The model is represented in panel A) of Figure 2. To assess the impact of the spatial distribution of ACh release, we built another 3D model in which 8% of the nodes were uniformly randomly selected all over the atria to be ACh release nodes. This model is represented in Figure 2B), and referred to as $D_{08}$ .

To build two additional models, we introduced a new hypothesis: the existence of minor branches that extend from the GPs and main nerves (Pauza et al., 2000; Fedele and Brand, 2020). We simplified this assumption by selecting nodes randomly, with percentages decreasing gradually as one moves radially away from the GPs and main nerves. Starting from the $O_{08}$ , additional nodes all over the atria were identified as release nodes with a probability that decreased with the distance to the octopus. The resulting percentage of ACh release node was about 30% and the model was consequently denoted as $O_{30}$ . Finally, to generate the $D_{30}$ model, 30% of all nodes in the atria were uniformly randomly selected as ACh release nodes. The $O_{30}$ and $D_{30}$ models are represented in Figures 2C, D, respectively.

In 2D, we defined two models of ACh release. In these models, denoted as $D_{2D,08}$ and $D_{2D,30}$ , respectively, we uniformly randomly selected 8% and 30% of the tissue to be ACh release nodes, similarly to the ACh diffuse 3D models.

Table 1 reports a summary of the different ACh release configurations.

**TABLE 1:** Summary of the different ACh release configurations.

	ACh release areas	Percentage of ACh release nodes
$O_{08}$	GP + Main nerves	8% of total number of nodes
$D_{08}$	Uniformly randomly distributed	8% of total number of nodes
$O_{30}$	GP + Main nerves + Smaller nerves	30% of total number of nodes
$D_{08}$	Uniformly randomly distributed	30% of total number of nodes
$D_{2D,08}$	Uniformly randomly distributed	8% of total number of nodes
$D_{2D,30}$	Uniformly randomly distributed	30% of total number of nodes

For both 2D and 3D models and for each of the identified release nodes, ACh was simulated to cyclically vary in time following a sinusoidal waveform of frequency equal to 0.125 Hz, which corresponds to the controlled respiratory frequency of the clinical recordings. We tested different mean concentrations of ACh $\left(\bar{ACh}\right)$ , equal to 0.05 and 0.075 *μ*M, and different peak-to-peak variations of ACh (ΔACh), equal to 0, 0.05 and 0.1 *μ*M. All simulated ACh values are within the physiological limits tested in preceding studies (0–0.1 *μ*M) (Bayer et al., 2019).

Furthermore, to investigate potential frequency-dependent behaviors and differences in modulation patterns, additional simulations were conducted. Additional frequencies of 0.20 and 0.33 Hz were specifically tested in 2D tissues and in 3D biatrial models stimulated with a train of ectopic beats. The $D_{2D,30}$ model and the $D_{30}$ model with ACh varying from 0 to 0.1 *μ*M were considered.

### 2.3 Numerical methods and simulations

Electrical propagation in the atria was described by the monodomain model and solved by means of the finite element method in combination with the operator splitting numerical scheme using the software ELVIRA (Heidenreich et al., 2010).

Single cells were paced at a fixed cycle length (CL) of 800 ms for 16 min to reach steady state. For 2D and 3D simulations, the state variables of the AP models were initialized to the steady-state values determined from single cell simulations.

Both the 2D and 3D models were pre-paced by delivering 14 stimuli at a CL of 800 ms. In the 2D tissues, the stimuli were applied onto the bottom edge of the tissue. In the 3D models, the stimuli were applied onto the region of the sinus node.

In the 2D tissue models, an S1-S2 cross-stimulation protocol was applied to initiate a rotor: the first stimulus (S1) was applied at the bottom edge of the tissue and the second stimulus (S2) was applied onto a 3.5 × 3.5 cm square at the bottom right corner.

In the 3D whole-atria models, two protocols were applied to initiate arrhythmias. The first one was an S1-S2 protocol similar to the one applied onto the 2D tissue, with the S1 stimulus delivered at a line joining the region between the superior and inferior left PVs and the region between the right PVs, and the S2 stimulus being subsequently applied parallel to the first one starting from the inferior left PV and covering only half of the S1 line length. The second protocol aimed to recreate a physiological setting and consisted in the application of an S1 stimulus followed by a train of premature stimuli delivered at a region surrounding the PVs, where ectopic beats are usually generated. In total, 10 stimuli were applied, with the first interval between stimuli being 200 ms and these intervals decreasing in 10-ms steps. Supplementary Figure S3 displays the delivery locations for S1 and S2 stimuli (panel (A)) and the delivery location of the ectopic beats (panel (B)).

### 2.4 Dominant frequency characterization

**FIGURE 3:**
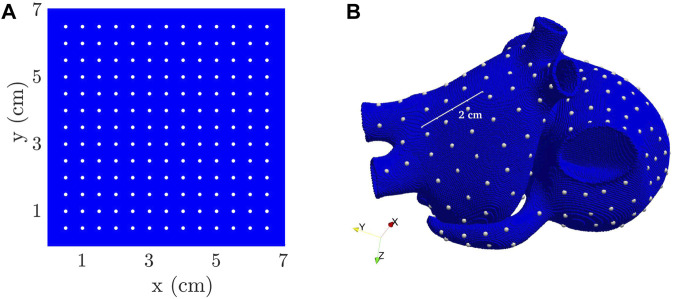
2D tissue **(A)** and 3D biatrial **(B)** models, with white dots representing the points used for the computation of *F*_f_.

 From the simulations, voltage time series were extracted from 169 evenly sampled points in the 2D tissue models and 223 points manually selected to be approximately uniformly distributed in the 3D whole-atria models (white dots in Figures 3A, B).

**FIGURE 4:**
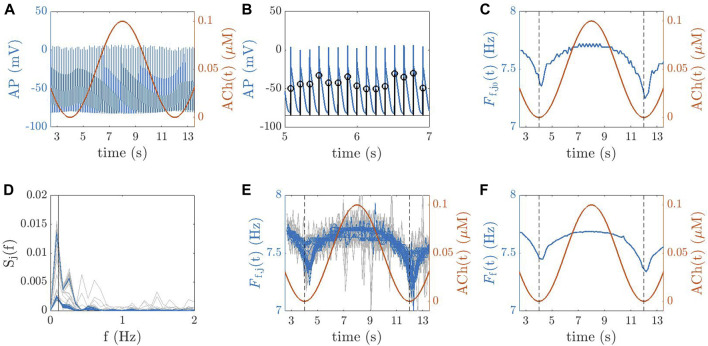
**(A)** AP trace for a point *j*_0_ in a 2D tissue (blue line) and ACh temporal variation, ACh(*t*) (red line). **(B)** Identification of the time instants for each beat *i*, denoted as *t*_*m*_ (*j*_0_, *i*), corresponding to the maximum AP upstroke velocity for the point *j*_0_. **(C)** Instantaneous frequency $F_{\text{f},j_{0}}\left(t\right)$ (blue line) and ACh(*t*) (red line). **(D)** “Peak-conditioned” selection of spectra computed for all spatial points *j* in the tissue. The discarded and accepted spectra are shown in grey and blue, respectively. **(E)** Instantaneous frequencies *F*_f,*j*_(*t*) for all spatial points *j* (blue and grey lines) and ACh(*t*) (red line). **(F)** Time series of the dominant frequency for the tissue, *F*_f_(*t*), obtained by spatial trimmed averaging (blue line) plotted on top of ACh(*t*) (red line).

**FIGURE 5:**
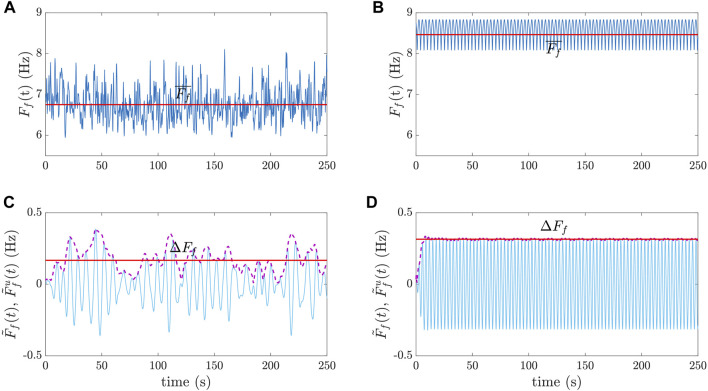
Examples of ${\bar{F}}_{f}$ and Δ*F*_f_ computation for clinical **(A)** and simulated **(B)** time series *F*_f_(*t*). The time series in **(B)** was obtained by time replication of an 8-s simulated signal. The corresponding bandpass-filtered time series ${\tilde{F}}_{f}\left(t\right)$ and their upper envelopes ${\tilde{F}}_{f}^{u}\left(t\right)$ , from which Δ*F*_f_ was computed, are displayed in panels **(C)** and **(D)**.

The AP trace for a point *j*_0_ in a 2D tissue is illustrated in Figure 4A. For each point *j* in each 2D tissue or 3D anatomy, the time instant *t*_*m*_ (*j*, *i*) corresponding to the maximum AP upstroke velocity of the *i*th beat was determined (Figure 4B). The instantaneous frequency was computed as *F*_f,j_ (*t*_*m*_ (*j*, *i*)) = 1/(*t*_*m*_ (*j*, *i*) − *t*_*m*_ (*j*, *i* − 1)) and linearly interpolated at 20 Hz to obtain *F*_f,j_(*t*), which is depicted in Figure 4C for point *j* = *j*_0_. Next, averaging (in space) was performed to compute the tissue dominant frequency *F*_f_(*t*) along time, as follows. First, each *F*_f,j_(*t*) was subjected to power spectral analysis to obtain *S*_*j*_(*f*). Spectral “peak-conditioned” selection was performed following the method described in Bailón et al. (2006) and the time series whose spectra were not sufficiently peaked were discarded (Figures 4D, E). *F*_f_(*t*) was eventually computed as the spatial mean of the remaining time series (Figure 4F). Finally, ${\bar{F}}_{f}$ was computed as the average value over time of *F*_f_(*t*) (Figures 5A, B), and the magnitude of f-wave frequency modulation, denoted as Δ*F*_f_, was computed by first bandpass filtering the time series *F*_f_(*t*) in a narrow frequency band (0.06 Hz) centered around the respiration rate, thus generating ${\tilde{F}}_{\text{f}}\left(t\right)$ , and subsequently computing its upper envelope ${\tilde{F}}_{\text{f}}^{u}\left(t\right)$ as the magnitude of its analytic equivalent using Hilbert transformation. The median over time of ${\tilde{F}}_{\text{f}}^{u}\left(t\right)$ was defined as Δ*F*_f_ (Abdollahpur et al., 2021), as illustrated in Figures 5C, D. In Table 2, ${\bar{F}}_{f}$ and Δ*F*_f_ are reported for the different simulations.

**TABLE 2:** ${\bar{F}}_{f}$ and Δ*F*_f_ (Hz) computed from simulations and from patients’ ECGs. Values are not reported for Δ*ACh* = 0.1 *μ*M and $\bar{ACh}$ = 0.075 *μ*M because the corresponding ACh values fell out of the physiological range.

		ΔACh [*μ*M]			ΔACh [*μ*M]		
ACh release 0.125 Hz		0.0	0.05	0.1	0.0	0.05	0.1
**2D SIMULATIONS**							
$\bar{ACh}$ [*μ*M]		$D_{2D,08}$			$D_{2D,30}$		
**0.05**	${\bar{F}}_{f}$	8.23	8.57	8.38	8.22	9.04	8.56
	Δ*F*_f_	0.00	0.05	0.10	0.00	0.13	0.30
	*ρ*		0.88	0.88		0.88	0.88
**0.075**	${\bar{F}}_{f}$	8.49	8.57	-	9.18	9.05	-
	Δ*F*_f_	0.00	0.04	-	0.00	0.15	-
	*ρ*		0.87	-		0.85	-
**3D S1-S2 SIMULATIONS**							
		$O_{08}$			$D_{08}$		
**0.05**	${\bar{F}}_{f}$	7.69	7.66	7.66	7.43	7.42	7.63
	Δ*F*_f_	0.00	0.01	0.11	0.00	0.01	0.08
	*ρ*		0.80	0.98		0.94	0.96
**0.075**	${\bar{F}}_{f}$	7.43	7.73	-	7.51	7.76	-
	Δ*F*_f_	0.01	0.02	-	0.00	0.01	-
	*ρ*		0.94	-		0.89	-
		$O_{30}$			$D_{30}$		
**0.05**	${\bar{F}}_{f}$	8.86	8.92	8.04	8.87	8.33	8.30
	Δ*F*_f_	0.00	0.04	0.19	0.00	0.12	0.35
	*ρ*		0.96	0.66		0.77	0.64
**0.075**	${\bar{F}}_{f}$	8.96	8.89	-	7.84	7.70	-
	Δ*F*_f_	0.00	0.03	-	0.00	0.03	-
	*ρ*		0.94	-		0.96	-
**3D ECTOPIC SIMULATIONS**							
		$O_{08}$			$D_{08}$		
**0.05**	${\bar{F}}_{f}$	5.18	5.19	5.21	5.03	5.11	5.18
	Δ*F*_f_	0.000	0.005	0.011	0.000	0.015	0.050
	*ρ*		-0.76	-0.69		-0.95	-0.95
**0.075**	${\bar{F}}_{f}$	5.18	5.18	-	4.97	4.97	-
	Δ*F*_f_	0.000	0.001	-	0.000	0.022	-
	*ρ*		-0.57	-		-0.93	-
		$O_{30}$			$D_{30}$		
**0.05**	${\bar{F}}_{f}$	4.52	4.76	4.99	4.60	4.74	4.94
	Δ*F*_f_	0.000	0.055	0.150	0.000	0.066	0.156
	*ρ*		-0.92	-0.97		-0.95	-0.98
**0.075**	${\bar{F}}_{f}$	4.62	4.66	-	4.63	4.65	-
	Δ*F*_f_	0.000	0.062	-	0.000	0.047	-
	*ρ*		-0.91	-		-0.90	-

MEAN VALUES						
	Clinical data (CR)	$D_{2D,08}$ , $D_{2D,30}$	$O_{08}$ , $D_{08}$ , S1-S2	$O_{30}$ , $D_{30}$ , S1-S2	$O_{08}$ , $D_{08}$ , Ect	$O_{30}$ , $D_{30}$ , Ect
${\bar{\bar{F}}}_{f}$	6.93	8.62	7.59	8.47	5.12	4.77
$\sigma_{{\bar{F}}_{f}}$	0.73	0.34	0.13	0.49	0.09	0.15
${\bar{\Delta F}}_{f}$	0.18	0.13	0.04	0.13	0.02	0.08
$\sigma_{\Delta F_{f}}$	0.09	0.09	0.04	0.12	0.02	0.04

The bold values represent the mean ACh level (0.05 or 0.075) on the first column, and the different values for Delta ACh (0.0, 0.05, and 0.1) on the second line.

### 2.5 Clinical recordings

By modeling and simulation of human atrial electrophysiology, we aimed at reproducing variations in *F*_f_ like those described in previous clinical studies (Holmqvist et al., 2005; Abdollahpur et al., 2021). A group of eight patients with psAF, atrioventricular block III and a permanent pacemaker was studied to investigate the modulation of *F*_f_ by respiration and its parasympathetic regulation. The study was conducted in accordance with the Declaration of Helsinki, and the protocol was approved by the local Ethics Committee. All subjects gave their informed consent for inclusion before they participated in the study. ECGs were recorded at rest during baseline (spontaneous respiration) (B) , during 0.125 Hz frequency-controlled respiration (CR) and during controlled respiration post atropine injection (PA) which led to full vagal blockade (Holmqvist et al., 2005). From the ECGs of the patients, the f-wave signal was obtained by applying spatiotemporal QRST cancellation (Stridh and Sörnmo, 2001). The *F*_f_ time series, denoted as *F*_f_(*t*), was estimated using a model-based approach (Henriksson et al., 2018), as previously described (Abdollahpur et al., 2021). ${\bar{F}}_{f}$ and Δ*F*_f_ were computed as explained in the previous section for the computational signals.

### 2.6 Statistical methods

From the simulations, results will be presented in terms of ${\bar{F}}_{f}$ and Δ*F*_f_, computed as described in section 2.4. While the range of ACh is considered from peak to peak amplitude, Δ*F*_f_ accounts for half of the span of the spectrum bandwidth computed from the upper envelope of the bandpass-filtered instantaneous frequency signal. This choice (i.e., considering half of the signal’s amplitude rather than the peak-to-peak amplitude) was made to be consistent with the clinical results presented in Abdollahpur et al. (2021). Mean values ( ${\bar{\bar{F}}}_{f}$ and ${\bar{\Delta F}}_{f}$ ) and standard deviations ( $\sigma_{{\bar{F}}_{f}}$ , $\sigma_{\Delta F_{f}}$ ) of ${\bar{F}}_{f}$ and Δ*F*_f_ were computed for each simulation, namely, the simulated cases using the 2D tissue model, 3D biatrial model stimulated with the S1-S2 protocol and 3D biatrial model stimulated with the ectopic beat protocol, with the 3D simulated cases separated into the 8% and 30% ACh release nodes cases.

From the patients, ${\bar{F}}_{f}$ and Δ*F*_f_ were computed for the B , CR and PA phases and the mean ± standard deviation over patients were reported following (Abdollahpur et al., 2021). These values are presented in Supplementary Table S2. From the CR phase, the mean over patients of ${{\bar{F}}_{f}}^{CR}$ , denoted as ${{\bar{\bar{F}}}_{\text{f}}}^{CR}$ , and of ${\Delta F_{f}}^{CR}$ , denoted as ${{\bar{\Delta F}}_{f}}^{CR}$ , along with the standard deviation over patients of those two measures, $\sigma_{{\bar{F}}_{f}^{CR}}$ and $\sigma_{\Delta F_{f}^{CR}}$ , respectively, were computed for comparison with the simulation results.

To evaluate the correlation between the variation in ACh concentration over time (ACh(*t*)) and the corresponding fibrillatory frequency signal *F*_f_(*t*) in the simulations, we used Spearman correlation coefficient (*ρ*).

In the clinical setting, the significance of the variations in the magnitude of the frequency modulation Δ*F*_f_ and in ${\bar{F}}_{f}$ between the B , CR and PA phases was tested and reported in Supplementary Table S2. Since the assumptions of normality and equal variances were not met, a Kruskal–Wallis test with Dunn-Sidak correction was employed.

## 3 Results

### 3.1 Clinical results

In all patients, the ECGs showed an f-wave frequency modulation in the B , CR and PA phases. In the CR phase, the magnitude of the frequency modulation was 0.18 ± 0.02 Hz. The magnitude of these variations was significantly reduced (*p* < 0.05) in 4 out of 8 patients after atropine-induced parasympathetic inhibition, while in the other four patients no changes were observed in the PA phase (Abdollahpur et al., 2021). Values from clinical recordings are reported in the bottom panel of Table 2 and in Supplementary Table S2, as described more in detail in section 2.6.

### 3.2 2D simulation results

**FIGURE 6:**
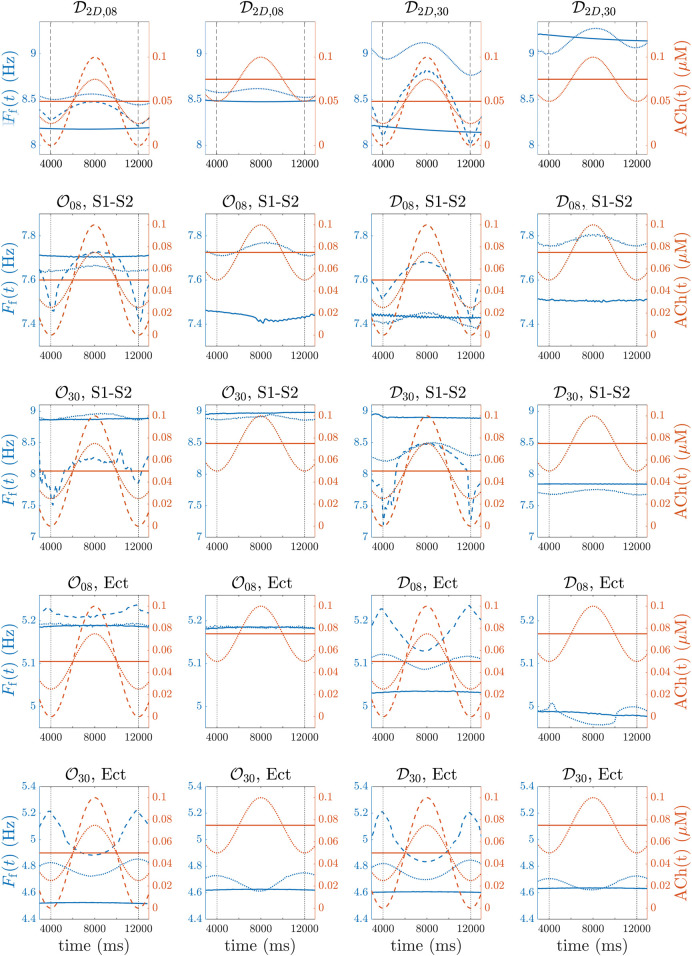
(first row, **(A–D)**), 3D biatrial simulations with $O_{08}$ and $D_{08}$ (second row, panels **(E–H)**) and $O_{30}$ and $D_{30}$ (third row, **(I–L)**) spatial configurations of ACh release and application of a S1-S2 protocol, 3D biatrial simulations with $O_{08}$ and $D_{08}$ (fourth row, **(M–P)**) and $O_{30}$ and $D_{30}$ (fifth row, **(Q–T)**) spatial configurations of ACh release and application of a train of ectopic beats. *F*_f_(*t*) (blue) and ACh(*t*) (red) are plotted in all panels for $\bar{ACh}=0.05\;\mu$ M **(A, C, E, G, I, K, M, O, Q, S)** and $\bar{ACh}=0.075\;\mu$ M **(B, D, F, H, J, L, N, P, R, T)**. Solid/dotted/dashed lines represent ΔACh values of 0.0/0.05/0.1 *μ*M. In panels **(B,D)**, ΔACh = 0.1 *μ*M was not included, as ACh(*t*) contained non-physiological ACh values.

Results in terms of mean dominant frequency ${\bar{F}}_{f}$ and Δ*F*_f_, as well as their mean and standard deviation between the different 2D cases, are reported in Table 2. In the 2D tissue models, a stable rotor was initiated after application of the S1-S2 protocol in both the $D_{2D,08}$ and $D_{2D,30}$ spatial configurations of ACh release. Simulation results in the 2D tissues confirmed that the rotor frequency variations followed the induced ACh patterns (Figure 6, first row), with Spearman correlation coefficient *ρ* between ACh(*t*) and *F*_f_(*t*) being above 0.85 in all cases, as reported in Table 2. Additionally, plots in the ACh-*F*_f_(*t*) plane have been provided in FIG SS in the Supplementary Material, particularly, results for the 2D cases are reported in the first row. Furthermore, *ρ* increased with $\bar{ACh}$ and ΔACh. ${\bar{F}}_{f}$ was found to be dependent on the $\bar{ACh}$ level, while Δ*F*_f_ was dependent on ΔACh, with increases in $\bar{ACh}$ and ΔACh leading to increases in ${\bar{F}}_{f}$ and Δ*F*_f_, respectively. It can be observed that ${\bar{F}}_{f}$ exhibits a non-monotonic dependence on ΔACh. Specifically, it appears that ${\bar{F}}_{f}$ is not solely determined by the $\bar{ACh}$ value, but also nonlinearly influenced by the combination of the minimum ACh level and ΔACh.

### 3.3 3D simulation results under S1-S2 stimulation

**FIGURE 7:**
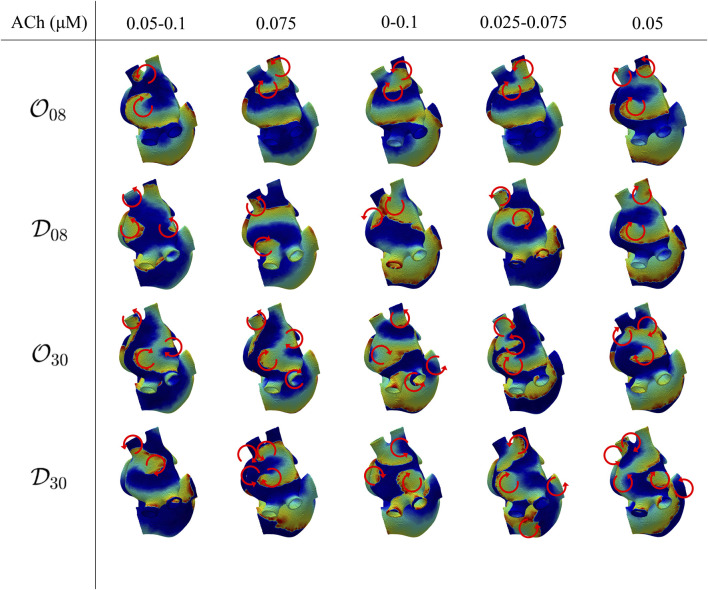
Voltage maps representative of the induced fibrillatory pattern after application of S1-S2 stimulation. Each circular arrow represents the location of a rotor with its direction of rotation. The ACh values indicate a constant ACh release when only a number is reported, corresponding to $\bar{ACh}$ , and to a range of variation when two numbers are given, corresponding to $\bar{ACh}\pm \Delta ACh$ .

In the 3D biatrial models, S1-S2 stimulation was able to generate multiple stable rotors. The induced fibrillatory patterns were different for the distinct spatial configurations of ACh release, with the generation of 2–5 stable rotors, as represented in Figure 7. We observed a general increase in the number of stable rotors with the highest percentage of ACh release nodes ( $O_{30}$ and $D_{30}$ ). In all cases, the rotors stabilized in the LA, with only four exceptions: $O_{30}$ model and $D_{30}$ model with ACh varying from 0.0 to 0.1 *μ*M, $D_{30}$ model with ACh varying from 0.025 to 0.075 *μ*M and $D_{30}$ model with ACh equal to 0.05 *μ*M, for which 2 to 4 stable rotors in the LA and 1 to 2 stable rotors in the RA were observed.

Similarly to the observations in the 2D tissues, for the four spatial ACh release models, i.e., $O_{08}$ , $D_{08}$ , $O_{30}$ and $D_{30}$ , the atrial dominant frequency followed the induced ACh(*t*) patterns (Figure 6, second and third rows and Supplementary Figure S4), with Spearman correlation coefficient values going from a minimum of 0.64 to a maximum of 0.96.

Table 2 shows ${\bar{F}}_{f}$ and Δ*F*_f_ for each of the simulated cases, as well as their mean and standard deviation between the aformentioned cases. Δ*F*_f_ was found to be highly dependent on ΔACh. Furthermore, for 30% ACh release nodes, Δ*F*_f_ was higher in the diffuse than in the octopus configuration. For 8%, the spatial distribution of ACh release did not have significant effects on Δ*F*_f_ when all other factors were kept constant. We observed that when the fibrillatory patterns in the 3D models were similar to one another, as occurs for the $O_{08}$ and $D_{08}$ models with ΔACh = 0.05 *μ*M, or for the $D_{08}$ model with ΔACh = 0.0 *μ*M, our results were in accordance with those in 2D tissues, with ${\bar{F}}_{f}$ increasing with $\bar{ACh}$ . As expected, we also observed an increase in the dominant f-wave frequency with the number of stable rotors in the atria regardless the $\bar{ACh}$ level. The octopus O and diffuse D configurations did not seem to correlate to the induced fibrillatory pattern and did not cause significant variations in terms of ${\bar{F}}_{f}$ , at least for 8% ACh release nodes. For 30% ACh release nodes, significant differences in ${\bar{F}}_{f}$ could be observed in several cases (up to −12.5% when comparing $O_{30}$ with respect to $D_{30}$ under $\bar{ACh}$ = 0.075 *μ*M and ΔACh = 0 *μ*M) while little or no differences could be observed in other cases. Also in this case, we observed a non-monotonic dependence between ${\bar{F}}_{f}$ and ΔACh. Similarly, both the minimum ACh and ΔACh can potentially influence ${\bar{F}}_{f}$ , but determining the extent of their impact is further complicated by the presence of different fibrillation patterns.

### 3.4 3D simulation results under stimulation with a train of ectopic beats

**FIGURE 8:**
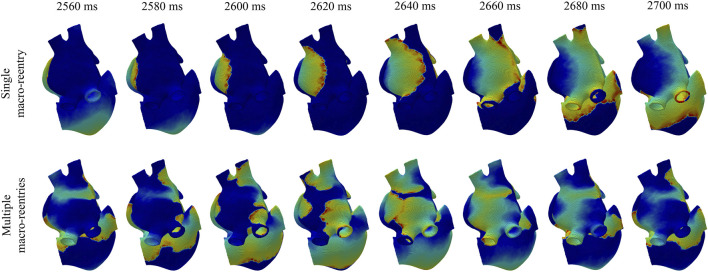
Voltage maps in a simulation where, after ectopic beat stimulation, a single macro-reentry was initiated (top panel) and in a simulation where, after S1-S2 stimulation, multiple macro-reentries were initiated (bottom panel).

When the 3D biatrial models were stimulated with a train of ectopic beats, a macro-reentry through the coronary sinus was generated in all cases. Figure 8, top row, illustrates voltage maps at different time instants in the case of a single macro-reentry (top-row) and in the case of multiple reentries generated by S1-S2 stimulation (bottom row) in the $D_{30}$ model, with ACh(*t*) varying from 0 to 0.1 *μ*M (ΔACh = 0.1 *μ*M). In this case, the frequency variations *F*_f_(*t*) were in opposite phase to the induced ACh(*t*) patterns (Figure 6, fourth and fifth rows, and Supplementary Figure S4). Table 2 reports ${\bar{F}}_{f}$ and Δ*F*_f_ for each of the simulated cases, along with their mean and standard deviation between the different cases. Also in this case, Δ*F*_f_ was greatly influenced by both ΔACh and the number of ACh release nodes. Moreover, ${\bar{F}}_{f}$ was observed to increase along with ΔACh. However, the relationship between ${\bar{F}}_{f}$ and $\bar{ACh}$ was not consistent across all cases. In some instances, there was a decrease, in others an increase, and in yet in others no change was observed.

For both 8% and 30% ACh release nodes, Δ*F*_f_ was higher in the diffuse than in the octopus configurations. Again, the different spatial distributions of ACh release, i.e., O and D configurations, did not cause significant variations in ${\bar{F}}_{f}$ . The absolute values of the Spearman correlation coefficient *ρ* were ranging between 0.57 and 0.98.

### 3.5 Dependency on ACh release frequency

When testing different ACh modulation frequencies (0.125/0.20/0.33 Hz), we found that, in the 2D cases, the rotor’s frequency consistently mirrored the induced ACh(*t*) frequency pattern. In the 3D cases, all tested ACh release frequencies led to reentry with the same frequency but opposite phase compared to the induced ACh(*t*) pattern. For both the single rotor case and the macro-reentry case, no substantial differences were observed in ${\bar{F}}_{f}$ and Δ*F*_f_ when changing the frequency of ACh release pattern. These results are presented in Supplementary Figure S5.

### 3.6 Comparison with clinical results

It can be noted that, when comparing simulated and clinical results, the mean f-wave frequency, ${\bar{F}}_{f}$ , was approximately 1–2 Hz higher in the simulations of both the 2D tissue models and the 3D biatrial models stimulated with the S1-S2 protocol than in the patients, while, in the 3D biatrial models stimulated with a train of ectopic beats, it was approximately 1–2 Hz lower. In the 3D models, the mean of Δ*F*_f_ values were lower than in the patients for models with 8% of ACh release nodes and close to the patients’ values for models with 30% of ACh release nodes.

## 4 Discussion

### 4.1 Characterization of f-wave frequency variations in response to cholinergic stimulation

We used computational modeling and simulation to assess the role of the ANS in the modulation of f-wave frequency. We built 2D tissue and 3D biatrial models in which we included different spatial distributions of ACh release. By additional modeling of the temporal evolution of cholinergic stimulation, we computed its impact on the atrial fibrillatory rate. We compared our simulation results with those from the analysis of patients’ ECGs, which showed that, in as much as 50% of the patients, the f-wave frequency modulation was strongly reduced after injection of the anticholinergic agent atropine. After confirmation of the agreement between simulation and clinical outcomes, we used our *in silico* approach to dissect the influence of spatiotemporal ACh release characteristics on f-wave frequency modulation independently of other factors.

For a comprehensive characterization of cholinergic effects on f-wave frequency modulation, we conducted simulations with different spatial configurations of ACh release, using a sinusoidal waveform with varying mean concentrations and peak-to-peak variation ranges of ACh. We also used two stimulation protocols: the S1-S2 protocol and a train of ectopic beats near the pulmonary veins in the 3D models.

The f-wave frequency *F*_f_(*t*) was determined by calculating the average of the instantaneous frequencies derived from several points evenly distributed across the tissue. These instantaneous frequencies were calculated as the reciprocals of the corresponding CLs. We also explored an alternate approach, wherein we initially calculated the mean CLs across the various points and subsequently computed *F*_f_(*t*) using the inverse of the mean CL along time. Nonetheless, since the discrepancies in CL values among the different points were negligible, the differences between the two approaches turned out to be negligible.

We found that the f-wave frequency responded to changes in ACh levels in both 2D tissue and 3D biatrial models. The average f-wave frequency was mainly influenced by the fibrillatory pattern, which was determined by the number and location of stable reentrant circuits. The percentage of ACh release nodes and the mean ACh concentration additionally contributed to the mean f-wave frequency. The peak-to-peak variation in the f-wave frequency was primarily influenced by the percentage of ACh release nodes and the temporal changes in ACh concentration. The distribution of ACh release sites, whether dispersed or in an octopus-like arrangement, had a minor impact.

Application of an S1-S2 protocol generated a single stable rotor in all 2D simulations while, in the 3D simulations, multiple stable reentries (2–5) were generated. In almost all the simulated cases, the generated rotors stabilized in the LA. Only in three cases, we observed stable rotors also in the RA. Rotor stabilization in the LA was expected, considering that we delivered the S1 and S2 stimuli in the region between the 4 pulmonary veins in the LA. The delivery of the stimuli in other atrial regions would be expected to lead to different fibrillatory patterns, with rotors stabilizing in other regions. Since we were interested in analyzing the ACh effects on the modulation of the atrial dominant frequency, our goal was to induce similar atrial activations in the different simulated cases to establish a comparison while reducing the influence of other factors. Nevertheless, we would not expect qualitatively different results when stimulating other atrial regions. When a train of ectopic beats was used to stimulate the atria, we observed a single macro-reentry in all cases. During both S1-S2 and ectopic stimulation, we did not include stimuli coming from the SAN to avoid more complex electrical activation patterns due to wavefront collision. The more stable patterns of atrial activation in our study allowed improved characterization of ACh effects over a period of 8 s.

The f-wave frequency trend *F*_f_(*t*) was computed and characterized with two variables: its temporal mean ${\bar{F}}_{f}$ , and the range of its modulation Δ*F*_f_. We found a clear correlation between the induced ACh(*t*) pattern and the computed *F*_f_(*t*) trend, with variations in *F*_f_(*t*) following the variations of ACh(*t*) throughout the atria, both for 8% and 30% of ACh release nodes. In the 2D tissues, where the fibrillatory activity consisted of a single stable rotor, we found that *F*_f_(*t*) varied in phase with ACh(*t*). In 3D biatrial anatomies, we found that *F*_f_(*t*) was in phase with ACh(*t*) when multiple reentries (AF pattern) were generated and was out of phase when a single macro-reentry (more similar to atrial flutter) was generated. A possible explanation could lie on the different relative contribution of CV and APD to the fibrillatory phenomena. For cases with a single macro-reentry, the main factor determining *F*_f_(*t*) modulation induced by ACh was CV. When ACh increased, the mean CV throughout the atria decreased and this led to a decrease in the rotation frequency of the macro-reentry. For cases with multiple stable rotors, *F*_f_(*t*) modulation was mainly determined by the shortening of the APD and ERP. For the highest ACh value, the APD and ERP were at its minimum and the rotor spun at an increased frequency due to the tissue being available earlier to be depolarized. To explore if the frequency variations observed during macro-reentry were linked to ACh-induced CV changes, we compared them with longitudinal CV values derived from 2D tissue simulations. Without ACh, the longitudinal CV was 50 cm/s, decreasing to 49.5 cm/s with 8% ACh relase nodes and further to 47.9 cm/s with 30% ACh release nodes (0.1 *μ*M ACh). In our biatrial models, considering that the macro-reentrant circuit measured approximately 14 cm, an increase in ACh concentration from 0 to 0.1 *μ*M resulted in frequency shifts of 0.01 Hz for $O_{08}$ and 0.1 Hz for $O_{30}$ . Correspondingly, CV changed from 73.14 to 72.90 cm/s for $O_{08}$ and from 73.28 to 71.81 cm/s for $O_{30}$ , consistent in magnitude with the 2D tissue results.

In this paper, we conducted frequency analysis using temporal traces of transmembrane potential instead of external electrical fields. To demonstrate that the same conclusions could be derived from ECGs, we carried out an additional analysis for selected cases in which we computed extracellular potentials and we conducted a frequency analysis on the resulting signals. Specifically, we considered four cases: $O_{08}$ and $O_{30}$ , with ACh concentrations ranging from 0 to 0.1 *μ*M, and we examined both the S1-S2 and ectopic beat excitation protocols. We calculated the equivalent dipole at 12 points located 3 cm away from the atrial tissue and arranged in a circular pattern around the atria. From these points, we obtained unipolar pseudo-ECG signals representative of the f-waves of the ECG. In Supplementary Figure S6, the left column displays a representative example of a simulated pseudo-ECG signal from one of the 12 points, while the right column represents the module of the Discrete Fourier transforms (DFT) of all the 12 lead signals (one for each of the points). For each case, we computed the mean of the frequencies corresponding to the largest peaks in the DFTs. This mean value represents a temporal mean of *F*_f_(*t*), denoted as ${\bar{F}}_{\text{f}}$ . We compared the computed results with those obtained from the transmembrane voltage (AP) traces, as reported in Table 2 of the manuscript, and we found them to be comparable, as presented in Supplementary Table S3.

Finally, in this study, we assumed that the activation of the ACh-activated potassium current occurred instantaneously following the release of ACh. However, it is important to note that there is a time delay, which is inversely proportional to both ACh concentration and temperature. In the literature (Inomata et al., 1989), latency times have been reported, ranging from 692 ± 50 ms for 0.01 *μ*M ACh to 98 ± 11 ms for 1 *μ*M ACh at 26°C. Additionally, variations in latency times from 267 ± 20 ms at 18°C to 44 ± 6 ms at 37°C for 1 *μ*M ACh have been observed as the temperature changes. In future studies, we may consider incorporating this dose-dependent time delay directly into the model to assess its impact on the results as ACh concentration varies. For the present analysis, we recalculated the Spearman coefficients between ACh(*t*) and *F*_f_(*t*) while introducing a fixed time delay of 420 ms for cases with a mean ACh concentration of 0.05 *μ*M and 350 ms for cases with a mean ACh concentration of 0.075 *μ*M. We did not observe any significant differences in the Spearman correlation coefficient when considering or omitting the time delay, as the delay was relatively small compared to the respiratory period. The maximum variation in the correlation coefficient was within ±0.10.

### 4.2 Contributors to mean f-wave frequency

${\bar{F}}_{f}$ was found to strongly depend on the fibrillatory pattern, generally increasing for a larger number of rotors. This finding was expected, considering that the larger the number of rotors, the smaller their size and the faster their spin. There were two exceptions to our findings, corresponding to the $O_{30}$ configuration with ΔACh of 0.0 and 0.05 *μ*M. Those cases showed a higher number of rotors under 0.075 *μ*M $\bar{ACh}$ , but ${\bar{F}}_{f}$ was equal or higher under lower $\bar{ACh}$ . A possible explanation could be based on the fact that, for the lower $\bar{ACh}$ , the rotors were closer in space, which led to smaller and faster spinning rotors. Conversely, in the other cases, while the number of rotors was higher, they were further apart from one another, resulting in larger and slower rotors.

The percentage of ACh release nodes in the atria emphasized the dependence of ${\bar{F}}_{f}$ on the fibrillatory pattern. A larger proportion of ACh release nodes led to higher ${\bar{F}}_{f}$ in the case of multiple reentries and lower ${\bar{F}}_{f}$ in the case of one single macro-reentry.

A third factor influencing ${\bar{F}}_{f}$ was $\bar{ACh}$ , which increased or decreased ${\bar{F}}_{f}$ in the same way as the percentage of $\bar{ACh}$ release.

The spatial distribution of ACh release nodes (diffuse or octopus) did not seem to be directly correlated to ${\bar{F}}_{f}$ . In most cases, no significant differences were observed between diffuse and octopus configurations, with only noticeable differences (up to 12%) found in specific cases, which could, however, be attributed to the fibrillatory pattern. Actually, for single macro-reentries, the differences in ${\bar{F}}_{f}$ between octopus and diffuse distributions were negligible. The higher differences were observed for the most chaotic AF patterns generated after S1-S2 stimulation in models with 30% ACh release nodes, where the fibrillatory pattern could have a larger impact. The negligible differences could be attributed to the passive space constant, which can lead to the averaging of electrical properties within the tissue.

Comparing simulation and clinical results in terms of ${\bar{F}}_{f}$ , we found higher ${\bar{F}}_{f}$ values for the models stimulated with an S1-S2 protocols and lower values for the models stimulated with a train of ectopic beats with respect to the clinical ${\bar{F}}_{f}$ values. In the simulations, the main factor determining ${\bar{F}}_{f}$ is the excitation pattern. For similar fibrillatory patterns, the differences in ${\bar{F}}_{f}$ could be attributed to $\bar{ACh}$ . The differences between simulation and clinical outcomes could be explained by the contribution of CV and the amount of fibrosis, on top of the values of $\bar{ACh}$ . Furthermore, the overestimation of ${\bar{F}}_{f}$ under S1-S2 stimulation may also depend on the APD of the cellular electrophysiological model. Indeed, the Courtemanche model used in this study has a steady-state APD that is at the lower end of the experimentally reported APD range.

### 4.3 Contributors to peak-to-peak variation in f-wave frequency

Δ*F*_f_ was found to be directly dependent on the percentage of ACh release nodes and on ΔACh. The spatial configuration of ACh release sites showed mild impact only for the highest (30%) percentage of ACh release nodes.

Comparing simulated and clinical values of Δ*F*_f_, better agreement was overall observed when considering 30% of ACh release nodes, either in a diffuse or octopus configuration. Higher values of ΔACh and larger percentages of ACh release sites along the atria could lead to simulated Δ*F*_f_ being closer to the values measured from clinical data.

### 4.4 Effects of ACh modulation frequency

In the simulations, a respiratory frequency of 0.125 Hz was assumed to match clinical results during controlled breathing. Nevertheless, additional simulations were conducted to investigate potential frequency-dependent behaviors and differences in modulation patterns. Additional frequencies of 0.20 and 0.33 Hz were specifically tested in 2D tissues to assess the impact of ACh release frequency on a single rotor in a scenario where variations in APD have a dominant impact on the WL, and in 3D biatrial models stimulated with a train of ectopic beats to evaluate the effects of ACh release frequency on a macro-reentry in a scenario where variations in CV have a dominant impact on the WL. The S1-S2 protocol was deliberately excluded due to complications arising from diverse fibrillatory patterns observed in such scenarios. From the results, it can be concluded that no qualitative or quantitative differences in *F*_f_ and Δ*F*_f_ were observed in the simulated time span when altering the frequency of ACh release frequency.

### 4.5 Comparison between simulations and clinical data

The inclusion of clinical data in our study was useful in determining a comprehensive range for the mean f-wave frequency and the magnitude of respiratory modulation. While not all our findings lie within this established range, there is some degree of overlap. The observed discrepancies could be attributed to various factors and can arise from limitations in either the clinical data or the simulations.

First, it is important to highlight the limited number of patients in the clinical dataset, with a large degree of variability over patients in terms of the mean and peak-to-peak variation of f-wave frequency. Furthermore, while in the simulations the modulation of the fibrillatory rate is determined only by ACh, in clinical signals variations in the respiratory frequency band can be influenced by factors other than the ANS. Mechanical stretch, mechano-electrical feedback or the endocrine system have been reported as possible modulators of the fibrillatory frequency (Gordan et al., 2015). These other mechanisms may interfere in a possibly nonlinear manner with the effects of ACh. Additionally, in the simulation setting, the use of a single anatomical model and a single model describing cellular electrophysiology may not have fully captured the inter-patient variability observed in the clinical scenario.

Considering that the clinical data we analyzed in our study was obtained from AF patients, we also compared our macro-reentry results with atrial flutter data from existing literature. A study conducted by Waxman et al. Waxman et al. (1991) reported atrial flutter CLs ranging from 185 to 350 ms (corresponding to frequencies of 5.40 to 2.86 Hz), with an average of 247.5 ms (corresponding to 4.04 Hz). It was also observed that the flutter CLs were consistently prolonged during inspiration and shortened during expiration, with an average respiratory excursion of 6.9 ms (corresponding to 0.11 Hz). In another study by Ravelli et al. (2008), the atrial flutter CL was reported to exhibit spontaneous beat-to-beat variability at the frequency of respiration. Consistent with the results described in Waxman et al. (1991), the respiratory modulation of atrial flutter CL displayed longer values during inspiration (223.2 ± 28.6 ms = 4.47 ± 0.49 Hz) and shorter values during expiration (221.1 ± 28.2 ms = 4.52 Hz ± 0.66 Hz). The frequency values reported in the atrial flutter studies align more closely with the values we observed in our macro-reentry simulations than with the AF values reported in Abdollahpur et al. (2021).

It is important to note that in the study by Ravelli et al., the observed oscillations in atrial flutter CL persisted even after pharmacologic autonomic blockade. As anticipated at the beginning of this section, this suggests the existence of factors independent of ANS that may contribute to respiratory-related atrial frequency oscillations. This could potentially explain the inter-patient variability observed in the clinical cases reported by Abdollahpur et al. Abdollahpur et al. (2021), where atropine reduced atrial frequency variability in just half of the patients. One of those additional factors could be the influence of mechanical stretch and mechano-electrical feedback. Different studies have shown that atrial tissue stretch affects atrial conduction and refractoriness in humans (Ravelli et al., 2008; Coronel et al., 2010), involving the activation of stretch-activated ion channels Sachs (1991); Hu and Sachs (1997). The mechanical regulation of heart rate has been proposed as a factor influencing RSA in heart transplant recipients (Bernardi et al., 1989; Bernardi et al., 1990), individuals with conditions associated with reduced vagal tone (El-Omar et al., 2001) and healthy individuals during exercise (Bernardi et al., 1990; Casadei et al., 1996). However, in normal physiological conditions, mechanical mechanisms are secondary to the effects of the ANS in modulating SAN pacemaker activity and, consequently, determining RSA (Kohl et al., 1999; Cooper and Kohl, 2005; Masè et al., 2009).

Extensive analysis has been conducted to understand the mechanical modulation, including cardiorespiratory modulation, of atrial activity, specifically in the case of atrial flutter (Waxman et al., 1991; Masé et al., 2008; Ravelli et al., 2008; Masè et al., 2009). Interventions like passive upright tilting, the strain phase of the Valsalva maneuver and expiration, all of which reduce cardiac size, have been observed to independently increased the rate of atrial flutter regardless of autonomic tone (Waxman et al., 1991). Finally, modeling studies have provided insights into the mechanoelectrical currents at the ionic level and their impact on AF (Kuijpers et al., 2007; Kuijpers et al., 2011). The magnitude of the stretch-activated current I_sac_ has been associated with the local stretch ratio and the heterogeneity in its activation has been shown to contribute to refractoriness dispersion and variation in conduction properties that increase the susceptibility to AF.

## 5 Study limitations and future work

Some limitations of this study should be acknowledged to provide direction for further work:

The clinical results are based on the method proposed in Abdollahpur et al. (2021), where all variations in the respiratory frequency interval are considered in the estimation of the respiratory modulation. Hence, variations in such frequency band that are unrelated to respiration may influence the result. In a more recent study, a subspace projection approach that only considers variations linearly related to respiration was proposed to quantify respiratory f-wave frequency modulation (Abdollahpur et al., 2022). However, the subspace projection approach could not be applied to the present dataset, since it requires a respiratory signal. In Abdollahpur et al. (2022), a respiratory signal was derived from the ECG, but the presence of pacemaker spikes in the present dataset makes that approach unfeasible.

Due to a lack of comprehensive understanding of the spatial distribution of parasympathetic innervation in the atria, we followed two approaches to represent ACh release nodes. One of such approaches is based on the octopus configuration reported in previous anatomical studies (Armour et al., 1997), which includes the spatial location of GPs and nerves departing from them. In the GPs, despite the predominance of parasympathetic fibers, sympathetic fibers can be found too (Tan et al., 2006). Here, we only considered cholinergic stimulation but future work could include additional modeling of the adrenergic stimulation.

We used the Courtemanche model to describe human atrial electrophysiology. Further investigations using other computational AP models with longer steady-state APD values could be conducted to assess the impact on f-wave frequency characterizations. Also, additional values of the longitudinal conductivity and the transverse-to-longitudinal conductivity ratio in the atrial tissue could be tested to identify the values leading to closer agreement between simulated and clinical f-wave frequency variables.

The computational models presented here were representative of psAF patients. Structural changes in the atria due to AF may present as various alterations such as an enlarged atrial chamber, hypertrophy of cardiomyocytes, increased mismatch between epicardial and endocardial myofibers’ orientations, changes in atrial wall thickness and, notably, an increased amount of fibrotic or connective tissue. Wyse et al. (2014); Schotten et al. (2011); Heijman et al. (2016). Fibrosis remodeling is a multiscale process associated with gap junction remodeling (Kostin et al., 2002; Brett et al., 2009), fibroblast proliferation (Rohr, 2009; Yue et al., 2011) and excess collagen deposition (Burstein and Nattel, 2008; Yue et al., 2011). We represented psAF-related structural remodeling by a combination of gap junction remodeling, modeled through tissue conductance reduction in fibrotic regions, and fibroblast proliferation. The latter was modeled by assigning some of the mesh nodes with fibroblast rather than myocyte properties and by using a fibroblast ionic model (MacCannell et al., 2007), as in previous studies (McDowell et al., 2013). Importantly, fibroblasts can exert electrophysiological influences on adjacent myocytes (Pedrotty et al., 2009) which can result in a reduction in APD, slower conduction and decreased excitability (Trayanova, 2014). Future studies including different degrees of fibrosis and other human atrial cell models, as well as different sites for external stimulation, could further investigate the impact of all these factors on ${\bar{F}}_{f}$ and Δ*F*_f_.

## 6 Conclusion

We assessed the impact of the spatiotemporal release pattern of ACh on f-wave frequency modulation. In agreement with clinical data, we found that the f-wave frequency varied in response to the temporal variation of ACh in both 2D tissue and 3D biatrial models. The mean f-wave frequency was found to be primarily dependent on the fibrillatory pattern, being largely determined by the number and spatial location of stable reentrant circuits. Other factors contributing to the mean f-wave frequency were the percentage of ACh release nodes throughout the atria and the mean ACh concentration. The peak-to-peak variation in the f-wave frequency was found to be dependent on the percentage of ACh release nodes and the variation over time in the ACh concentration. The spatial distribution of ACh release sites, either diffuse or following an octopus configuration, showed only mild impact.

## Data Availability

The raw data supporting the conclusion of this article will be made available by the authors, without undue reservation.
