# Supplementary material for: The frequency of atrial fibrillatory waves is modulated by the spatiotemporal pattern of acetylcholine release: a 3D computational study
Source: Front Physiol. 2024 Jan 3;14:1189464. doi: 10.3389/fphys.2023.1189464 (PMC10791938; doi:10.3389/fphys.2023.1189464)
Supplement: Supplementary file 1 [file DataSheet1.PDF]

Supplementary Material

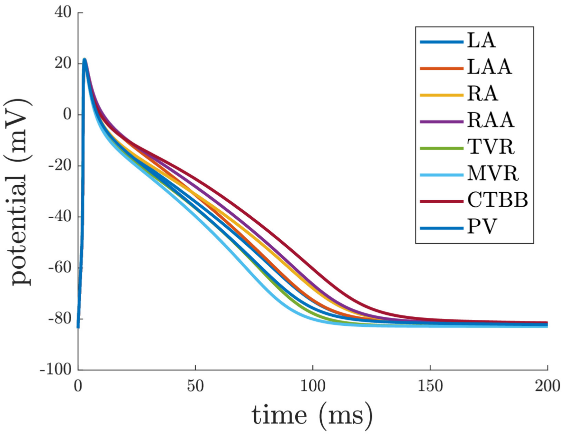

Figure S1. APs corresponding to the different electrophysiological regions in the 3D biatrial models.

Table S1. Longitudinal conductivity (L_{CV}) and transverse-to-longitudinal conductivity ratio (T/L_{CR}) values for the different atrial regions.

**Region**	$\mathbf{L_{CV}}$ **(S)**	$\mathbf{T/L_{CR}}$
RA	0.0030	0.35
CT	0.0085	0.15
PV	0.0017	0.50
BBPM	0.0075	0.15
IST	0.0015	1.00
SAN	0.0008	1.00
FO	0.0000	1.00
CS	0.0060	0.5
LA	0.0030	0.25
LFO	0.0075	0.15

REFERENCES

Abdollahpur, M., Holmqvist, F., Platonov, P. G., and Sandberg, F. (2021). Respiratory Induced Modulation in f-Wave Characteristics During Atrial Fibrillation. *Frontiers in Physiology* 12, 653492. doi:10.3389/fphys.2021.653492

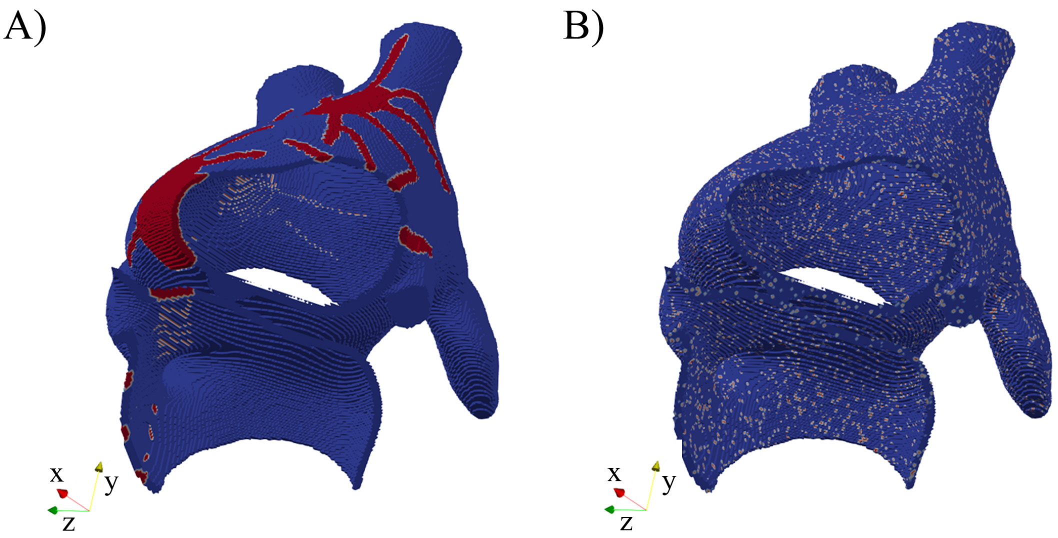

Figure S2. Sections of the left atrium for the $\mathcal{O}_{08}$ model (A) and $\mathcal{D}_{08}$ model (B). In the $\mathcal{O}_{08}$ the ACh release nodes (red) are concentrated in the 2 most external layers of the mesh, while in the $\mathcal{D}_{08}$ model they are uniform randomly distributed in all the three node layers.

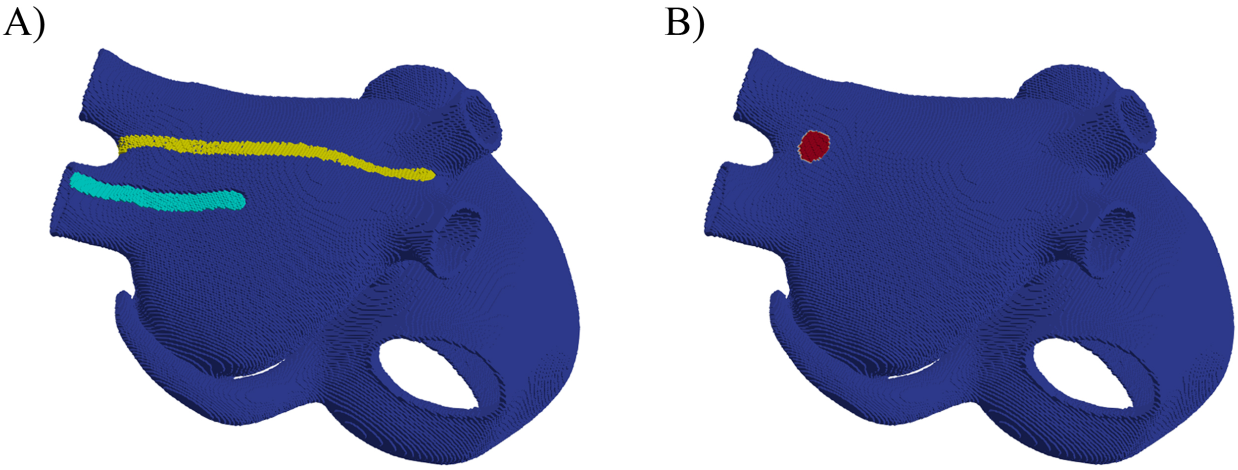

Figure S3. A) Location of the S1 stimulation (yellow region) and of the S2 stimulation (cyan region). B) Location of ectopic beats stimulation (red region)

Table S2. Results from clinical data ($\mathcal{B}$: baseline, $\mathcal{CR}$: Controlled Respiration, $\mathcal{PA}$: Post Atropine). ($*$) indicates significant differences to $\mathcal{B}$ and ($\bullet$) indicates significant differences to $\mathcal{CR}$. The quality of baseline signals of patient f was too low to evaluate $\Delta F_{\mathrm{f}}{}^{\mathcal{B}}$ and $\overline{F}_{\mathrm{f}}{}^{\mathcal{B}}$. Table adapted from Abdollahpur et al. (2021).

**Patient**	$\Delta F_{\mathrm{f}}{}^{\mathcal{B}}$ (Hz)	$\Delta F_{\mathrm{f}}{}^{\mathcal{CR}}$ (Hz)	$\Delta F_{\mathrm{f}}{}^{\mathcal{PA}}$ (Hz)	$\overline{F}_{\mathrm{f}}{}^{\mathcal{B}}$ (Hz)	$\overline{F}_{\mathrm{f}}{}^{\mathcal{CR}}$ (Hz)	$\overline{F}_{\mathrm{f}}{}^{\mathcal{PA}}$ (Hz)
a	0.16 ± 0.06	$0.17 \pm 0.09^{*}$	$0.16 \pm 0.08^{\bullet}$	6.84 ± 0.43	$6.67 \pm 0.45^{*}$	$6.71 \pm 0.41^{\bullet *}$
b	0.13 ± 0.06	$0.16 \pm 0.08^{*}$	$0.13 \pm 0.07^{\bullet}$	6.17 ± 0.35	$6.31 \pm 0.37^{*}$	$6.46 \pm 0.36^{\bullet *}$
c	0.16 ± 0.08	$0.17 \pm 0.08^{*}$	$0.14 \pm 0.07^{\bullet *}$	6.57 ± 0.43	$6.75 \pm 0.39^{*}$	$6.36 \pm 0.43^{\bullet *}$
d	0.16 ± 0.08	$0.19 \pm 0.10^{*}$	$0.19 \pm 0.10^{*}$	7.68 ± 0.51	$7.39 \pm 0.54^{*}$	$7.57 \pm 0.54^{\bullet *}$
e	0.14 ± 0.07	$0.21 \pm 0.10^{*}$	$0.21 \pm 0.12^{*}$	7.46 ± 0.46	$7.29 \pm 0.49^{*}$	$7.14 \pm 0.48^{\bullet *}$
f	-	0.17 ± 0.08	$0.16 \pm 0.08^{\bullet}$	-	5.91 ± 0.41	$5.96 \pm 0.42^{\bullet}$
g	0.15 ± 0.08	$0.17 \pm 0.07^{*}$	$0.17 \pm 0.07^{*}$	7.73 ± 0.43	$8.11 \pm 0.44^{*}$	$7.98 \pm 0.43^{\bullet *}$
	$\overline{\Delta F}_{\mathrm{f}}{}^{\mathcal{B}}$ (Hz)	$\overline{\Delta F}_{\mathrm{f}}{}^{\mathcal{CR}}$ (Hz)	$\overline{\Delta F}_{\mathrm{f}}{}^{\mathcal{PA}}$ (Hz)	$\overline{\overline{F}}_{\mathrm{f}}{}^{\mathcal{B}}$ (Hz)	$\overline{\overline{F}}_{\mathrm{f}}{}^{\mathcal{CR}}$ (Hz)	$\overline{\overline{F}}_{\mathrm{f}}{}^{\mathcal{PA}}$ (Hz)
Average	0.15 ± 0.01	0.18 ± 0.02	0.17 ± 0.03	7.07 ± 0.64	6.93 ± 0.73	6.88 ± 0.71

Table S3. Comparison of results obtained from the transmembrane voltage (AP) traces and from pseudo-ECGs, in terms of $\overline{F}_{\mathrm{f}}$.

	$\overline{F}_{\mathrm{f}}$ from AP (Hz)	$\overline{F}_{\mathrm{f}}$ from ECG (Hz)
$\mathcal{O}_{08}$ S1-S2	7.66	7.67
$\mathcal{O}_{08}$ Ect	5.21	5.25
$\mathcal{O}_{30}$ S1-S2	8.04	8.00
$\mathcal{O}_{30}$ Ect	4.99	5.01

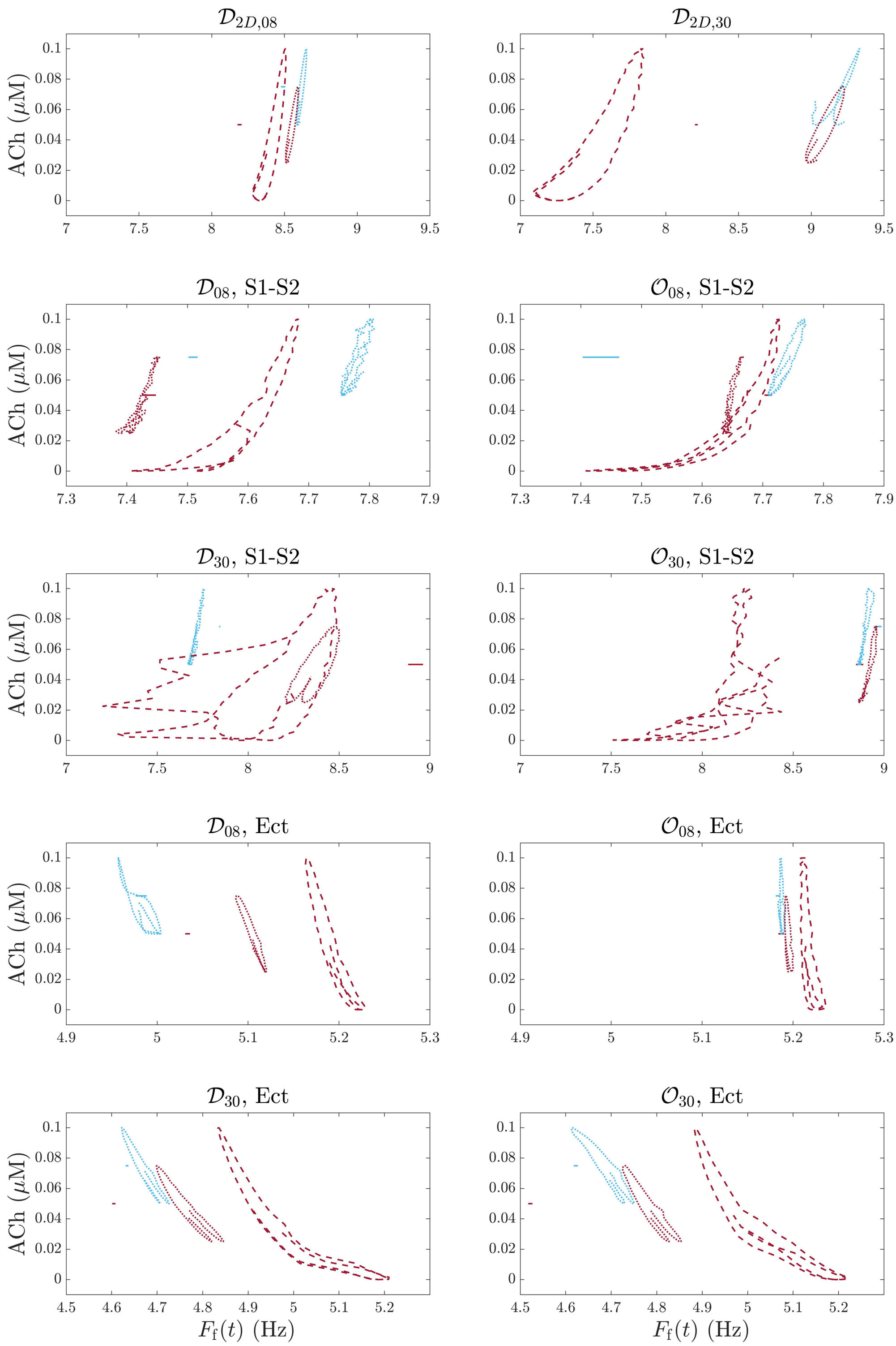

Figure S4. $F_{\mathrm{f}}(t)$ versus ACh(t) for $\mathcal{D}_{2D,08}$ and $\mathcal{D}_{2D,30}$ (first row), $\mathcal{D}_{08}$ and $\mathcal{O}_{08}$ (second row) and $\mathcal{D}_{30}$ and $\mathcal{O}_{30}$ (third row) and application of the S1-S2 protocol, $\mathcal{D}_{08}$ and $\mathcal{O}_{08}$ (fourth row) and $\mathcal{D}_{30}$ and $\mathcal{O}_{30}$ (fifth row) and application of a train of ectopic beats. Light blue/dark red represents $\overline{ACh}$=0.075/$\overline{ACh}$=0.05 μM. Solid/dotted/dashed lines represent ΔACh values of 0.0/0.05/0.1 μM.

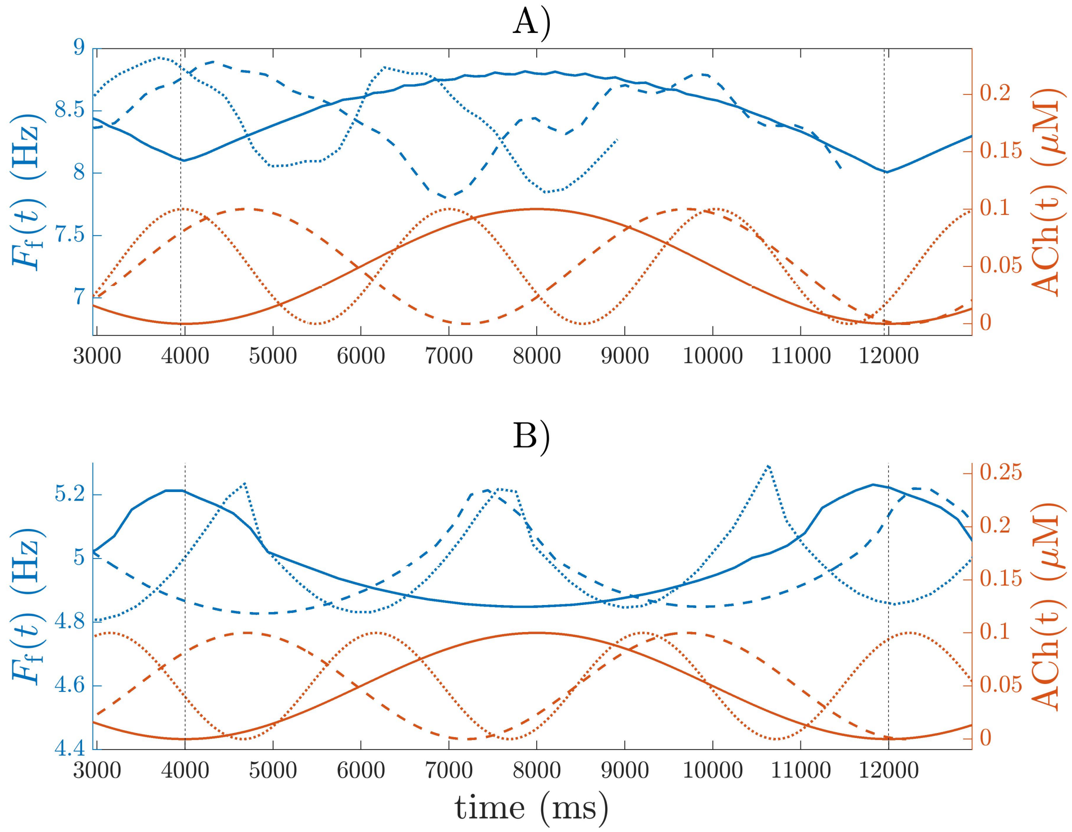

Figure S5. A) Results from 2D tissue simulations with $\mathcal{D}_{2D,30}$ spatial configurations of ACh release. B) Results from 3D biatrial simulations with $\mathcal{D}_{30}$ spatial configurations of ACh release and application of a train of ectopic beats. $F_{\mathrm{f}}(t)$ (blue) and $\mathrm{ACh}(t)$ (red) are plotted in both panels for $\overline{\mathrm{ACh}} = 0.1$ μM. Solid/dashed/dotted lines represent different ACh concentration modulation frequencies (0.125/0.20/0.33 Hz, respectively). In panel A), both the 0.20 Hz and 0.33 Hz frequencies of ACh modulation resulted in premature rotor extinction at the tissue borders.

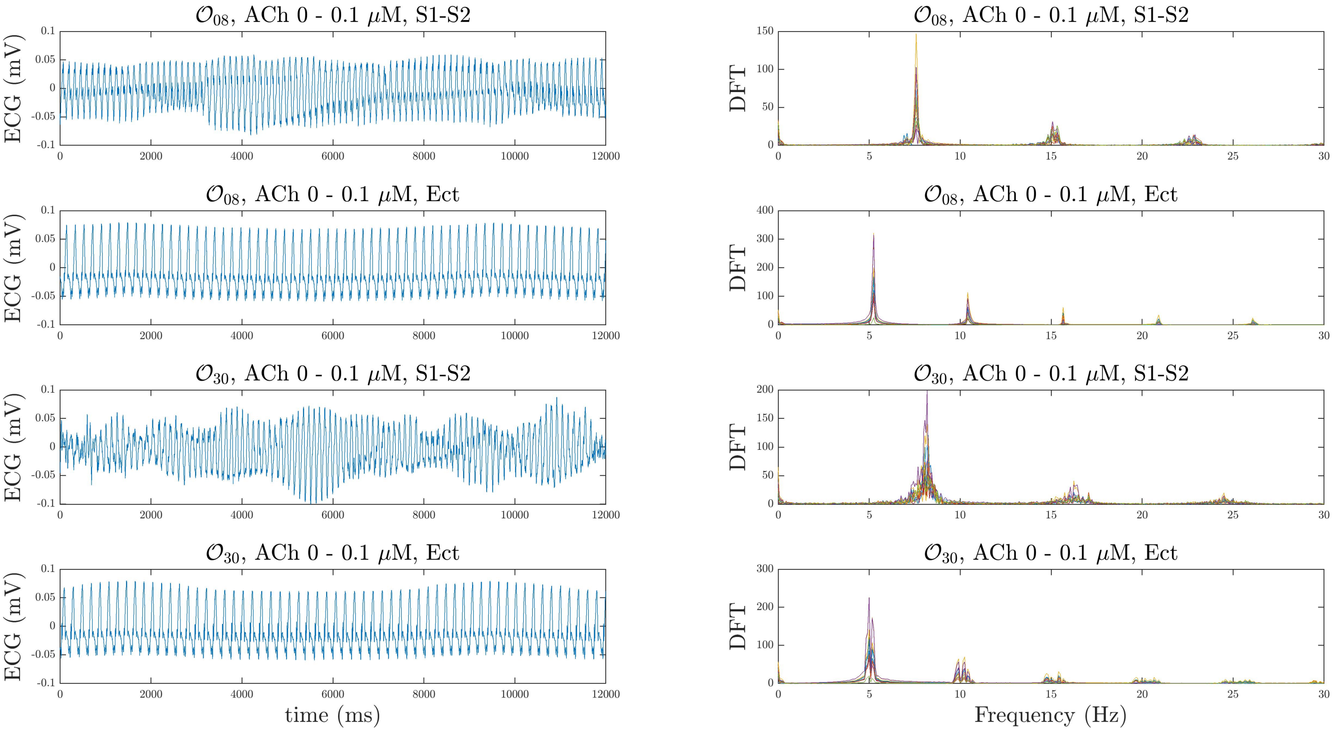

Figure S6. Left column) ECG signal from one of the 12 electrodes. Right column) Module of the Discrete Fourier transforms (DFT) of the 12 pseudo-ECG signals.
